# Clinical Cases

**Published:** 1884-04

**Authors:** J. D. Turrill

**Affiliations:** Port Clinton, Ohio


### CLINICAL BUREAU.

### CLINICAL CASES.

J. D. TURRILL, M. D., PORT CLINTON, OHIO.

In April, 1883, Mrs. G. R. H., æt. about fifty years, called at office to have right index finger amputated. Not practicing surgery, I declined to officiate, but gleaned the following history: One year ago she ran a fine splinter of glass in the apex of index finger and did not succeed in removing all the glass, as it became finely comminuted. Gave no trouble till early last summer, when the end of the finger became painful and very sensitive; this pain extended up the right arm to axilla, and thence radiated to right side of thorax; there were some lymphatic swellings and loss of power in the right extremity, threatening paralysis; she was unable to perform her household duties without pain, which was wearing her out. On consulting a pseudo-homœopathist she was told that her finger must be amputated, and to her friends he said he expected to have to remove her entire arm. The lady shrank from this ultimatum, and bore the pain till it was no longer endurable, when she summoned her fortitude and came to town, a distance of twelve miles, to undergo the operation, only to find the doctor absent; she then came to me, and after eliciting these facts I told her I felt sure she need not lose either her arm or finger if she were willing to try Homœopathy. She decided to give medicine a fair show; she was worn out in body and mind; face pale, cachectic; scrofulous diathesis; restless, fidgety; right arm and wrist weak; felt as if she could not lift or do anything; nails yellow and brittle; insomnia from pain; feels unrefreshed in morning. The balance of symptoms I cannot bring to mind, having inadvertently burned record with some waste paper. My choice of remedy was finally fixed by reading "small foreign bodies under skin; promotes expulsion," in *Hering's Condensed Materia Medica*, chapter 45, last line, under Silicea. ℞ Silicea 6$^{m}$ three powders one daily then placebos till May 2d.; when I found suppuration going on, end of finger being very callous. I applied some emollient to soften tissues; in a few more days some fine particles of glass came out; and during that month, and through June glass continued to be discharged; she had at intervals a feeling as if some small object were working its way down the arm; this would be followed in a few days by discharge of pus and glass; till about August she said she could trace one piece coming down; this came away, and since then general health has improved and she has resumed her domestic duties, which are arduous, and has no more pain or trouble in side, arm, or finger.

